# A case of unilateral dysmenorrhea

**DOI:** 10.4103/0974-1208.74162

**Published:** 2010

**Authors:** Tulon Borah, Ananya Das, Subrat Panda, Santa Singh

**Affiliations:** Department of Obstetrics and Gynecology, North Eastern Indira Gandhi Regional Institute of Health and Medical Sciences, Shillong, Meghalaya, India

**Keywords:** Rudimentary horn, unilateral dysmenorrhea, uterine malformation

## Abstract

Unilateral dysmenorrhea in an adolescent may be associated with uterine malformation. Relevant investigations in suspected cases and timely intervention can prevent future complications in such cases. Here, we present a case of unicornuate uterus with rudimentary horn in an adolescent complaining of unilateral dysmenorrhea.

## INTRODUCTION

Dysmenorrhea is a common complaint of adolescent girls attending the outpatient department and is also the most common reason for school absence among them. The prevalence of dysmenorrhea is estimated to be around 25% of all women and up to 90% of adolescents.[1] Secondary dysmenorrhea is of a greater concern, which is generally pathological in nature and requires treatment. The rare variety of unilateral dysmenorrhea in a young patient should always raise the suspicion of uterine malformation.

## CASE REPORT

**Figure 1:**
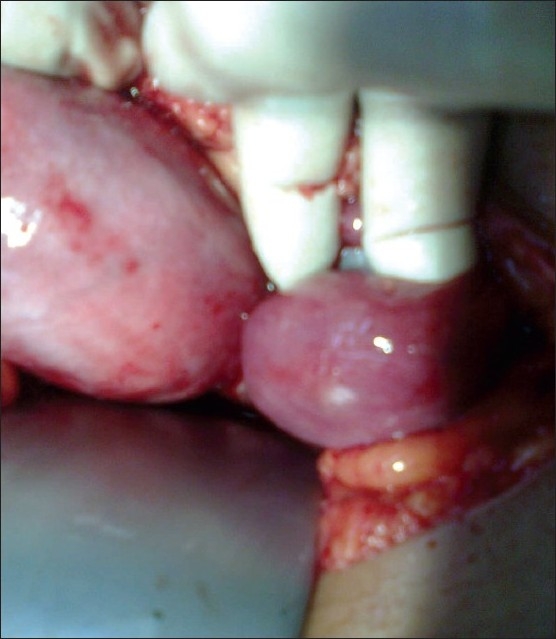
Unicornuate uterus with right-sided rudimentary horn

An 18-year-old girl attended to the outpatient department of our hospital on the sixth day of her menstrual cycle with pain. She had such complaints during menstrual cycle since the last 2 years. The pain was typically felt over the right lower abdomen and used to start just before the menstrual cycle, and reached its peak during menstrual cycle. She also gave a history of taking analgesics on and off for it. On examination, her vitals were stable and no abnormality was detected on systemic examination. On per-abdominal examination, there was tenderness at the right iliac fossa. She was also advised ultrasonography (USG) to rule out any pelvic pathology. USG suggested differential diagnoses of right-sided hematosalpinx, with hematometra and bicornuate uterus, with non-visualized right kidney. To confirm the diagnosis, magnetic resonance imaging (MRI) was performed, which showed a unicornuate uterus with right-sided functioning cavitary rudimentary horn, right hematometra and hematosalpinx, right ovarian hemorrhagic cyst and right renal agenesis. Intravenous pyelography also confirmed right renal agenesis. On urology consultation, cystoscopy was performed, which revealed non-visualization of the right ureteric orifice with normal left ureteric orifice and bladder mucosa. Even though laparoscopy is the preferred approach in such cases with available facility and expertise, we decided to proceed with laparotomy. On laparotomy, two separate cornu of uterus [Figure 1] with right-sided hematosalpinx and right ovarian hemorrhagic cyst were seen. On incising the right-sided horn of the uterus, anchovy sauce-like material came out. The right-sided rudimentary horn was found to be non-communicating with the main uterine cavity. Excision of the right-sided cornu and right ovariotomy was performed maintaining proper hemostasis. Left cornu and fallopian tube were preserved. The excised specimen was then sent for histopathological examination. Her post-operative period was uneventful. Histopathology revealed rudimentary horn with endometrial and myometrial tissue. The right-sided tube showed chronic salpingitis with areas of hemosiderin-laden macrophages and the ovary had features suggestive of simple hemorrhagic follicular cyst. The patient had attended our outpatient department for follow-up and had no complaint of dysmenorrhea.

## DISCUSSION

Unicornuate uterus with a rudimentary horn is a rare type of mullerian duct malformation, with a reported incidence of 0.06%.[2] In 83% of the cases, the rudimentary horn is non-communicating.[3] The rudimentary horn may consist of a functional endometrial cavity or it may be a small solid lump of uterine muscle with no functional endometrium. mullerian anomalies are commonly associated with absence or gross malformation of the renal tract. It is important to keep an index of suspicion in high-risk groups of uterine or mullerian anomalies regarding other common spinal, cloacal and renal anomalies.[4]

A unicornuate uterus causes few symptoms and it is usually discovered by chance or as a result of pregnancy complications. But, in our case, the patient presented with unilateral dysmenorrhea. When dysmenorrhea is with rudimentary horn, it is usually because of the obstruction to the outflow of menstrual blood. Other causes of unilateral dysmenorrhea may be endometriosis with unilateral distribution or a small leiomyoma at the uterotubal junction. One-sided spasmodic dysmenorrhea in a young girl should always raise the suspicion of uterine malformation, and every effort should be made to exclude the condition by conducting relevant investigations. The diagnosis of mullerian abnormalities can be made via USG. However, MRI is more specific for the evaluation of presence or absence of a functional endometrium. But, the gold standard to diagnose mullerian anomaly is diagnostic laparoscopy.

It is generally considered that the presence of a non-communicating cavitory rudimentary horn carries increased risks of endometriosis and cornual pregnancy. It is difficult to truly estimate the incidence of such complications as the data available are in the form of case reports, and they usually present as surgical emergencies. To avoid serious complications in the future, early diagnosis and excision of the rudimentary horn is of utmost importance. If facility and expertise are available, laparoscopic excision should be offered especially to the young girls as it is less morbid and cosmetically more acceptable.

## CONCLUSION

Unilateral dysmenorrhea, although unusual, might present as acute abdomen. It should be thoroughly evaluated especially in adolescents with relevant investigations keeping in mind the rare possibility of mullerian anomaly. Early diagnosis and prompt treatment is recommended in these cases to avoid future gynecological and obstetrical complications. The right step at the right moment aided with maximal suspicion and investigation and minimal access surgery is the preferred mode of management here.
